# Anti-inflammatory effects of N-acylethanolamines in rheumatoid arthritis synovial cells are mediated by TRPV1 and TRPA1 in a COX-2 dependent manner

**DOI:** 10.1186/s13075-015-0845-5

**Published:** 2015-11-14

**Authors:** Torsten Lowin, Martin Apitz, Sven Anders, Rainer H. Straub

**Affiliations:** Laboratory of Experimental Rheumatology and Neuroendocrine Immunology, Department of Internal Medicine I, University Hospital Regensburg, Franz Josef Strauss Allee 11, 93042 Regensburg, Germany; Department of Orthopaedic Surgery, University Hospital Regensburg, Asklepios Clinic Bad Abbach, Kaiser Karl V Allee 3, 93077 Bad Abbach, Germany

**Keywords:** Synoviocytes, Tumor necrosis factor, Interleukin 6, Interleukin 8, Synovial fibroblasts, TRPV1, TRPA1, Cannabinoids, Anandamide, Matrix metalloproteinase 3, Collagen-induced arthritis

## Abstract

**Introduction:**

The endocannabinoid system modulates function of immune cells and mesenchymal cells such as fibroblasts, which contribute to cartilage destruction in rheumatoid arthritis (RA). The aim of the study was to determine the influence of N-acylethanolamines anandamide (AEA), palmitoylethanolamine (PEA) and oleylethanolamine (OEA) on several features of arthritic inflammation in vitro (human material) and in vivo (a mouse model).

**Methods:**

Immunofluorescence and western blotting were used to detect cannabinoid receptors and related enzymes. Cytokines and MMP-3 were measured by ELISA. Intracellular signaling proteins were detected by proteome profiling. Proliferation was quantified by CTB reagent. Adhesion was assessed by the xCELLigence system. After onset of collagen type II arthritis, mice were treated daily with the FAAH inhibitor JNJ1661010 (20 mg/kg) or vehicle.

**Results:**

IL-6, IL-8 and MMP-3 (determined only in synovial fibroblasts (SFs)) were downregulated in primary synoviocytes and SFs of RA and OA after AEA, PEA and OEA treatment. In SFs, this was due to activation of TRPV1 and TRPA1 in a COX-2-dependent fashion. FAAH inhibition increased the efficacy of AEA in primary synoviocytes but not in SFs. The effects of OEA and PEA on SFs were diminished by FAAH inhibition. Adhesion to fibronectin was increased in a CB_1_-dependent manner by AEA in OASFs. Furthermore, elevation of endocannabinoids ameliorated collagen-induced arthritis in mice.

**Conclusions:**

N-acylethanolamines exert anti-inflammatory effects in SFs. A dual FAAH/COX-2 inhibitor, increasing N-acylethanolamine levels with concomitant TRP channel desensitization, might be a good candidate to inhibit the production of proinflammatory mediators of synovial cells and to reduce erosions.

**Electronic supplementary material:**

The online version of this article (doi:10.1186/s13075-015-0845-5) contains supplementary material, which is available to authorized users.

## Introduction

Rheumatoid arthritis (RA) is a chronic inflammatory disease characterized by cartilage and bone destruction [1]. Besides infiltration of lymphocytes into synovial tissue, hyperproliferation of synovial fibroblasts (SF), which produce matrix-degrading enzymes and proinflammatory cytokines, is an important propagator of RA [2]. SF migrate to distant sites and may thereby spread arthritis to unaffected joints [3]. Furthermore, due to the proinflammatory environment in the joint, hypoxia develops, which leads to the upregulation of a wide array of proinflammatory genes in RASF [4, 5]. Data from Richardson and colleagues suggest that the synovial endocannabinoid system is altered in RA. While the endocannabinoid arachidonylethanolamine (anandamide, AEA) and 2-archidonyl glycerol (2-AG) are absent from healthy joints, where related N-acylethanolamines oleoylethanolamine (OEA) and palmitoylethanolamine (PEA) are abundant, both 2-AG and AEA were found in joints of arthritic and osteoarthritic patients [6].

Endocannabinoids (EC) are neuromodulatory lipid mediators that exert their effects mainly by activating cannabinoid receptor type 1 (CB_1_) and type 2 (CB_2_) [7]. However, additional targets for EC and related N-acylethanolamines were identified. These include the transient receptor potential vanilloid channel TRPV1, peroxisome proliferator-activated receptors α and γ but also G protein-coupled receptors GPR18 and GPR55 [8–10]. Experiments demonstrated that some cannabinoid effects are attributed to activation of these receptors [10]. AEA, OEA and PEA but also 2-arachidonylglycerol (2-AG) are produced on demand from lipid precursors in the cell membrane [11]. Their action is limited by degradation by either monoacylglycerol lipase (MAGL, specific for 2-AG) or fatty acid amide hydrolase (FAAH, specific for AEA, OEA and PEA) although alternative routes of degradation exist. Pharmacological inhibition of FAAH or MAGL increases systemic levels of the respective EC [12]. Besides their well-characterized central effects, EC also reduce the production of proinflammatory cytokines in various cell types, decrease T cell proliferation and inhibit migration of immune cells [13].

Currently, the effects of N-acylethanolamines on production of inflammatory mediators in primary synoviocytes or SF have not been described. In this study, we investigate their effects on primary synoviocytes (AEA only) but also on SF from RA and osteoarthritis (OA). It is shown how AEA regulates tumor necrosis factor (TNF), interleukin-6 (IL-6), interleukin-8 (IL-8) and matrix metalloproteinase 3 (MMP-3) production, mitogen-activated protein (MAP) kinase signaling and SF adhesion. In addition, the involvement of cyclooxygenase-2 (COX-2), TRPV1 and transient receptor potential cation channel (TRPA1) in mediating the effects of AEA but also PEA and OEA is revealed, increasing possible therapeutic targets for the treatment of RA. Furthermore, it is demonstrated that systemic FAAH inhibition is beneficial in collagen type II-induced arthritis (CIA).

## Materials and methods

### Patients

In this study, 28 patients with long-standing RA fulfilling the American College of Rheumatology revised criteria for RA [14] and 56 patients with OA were included. The RA group comprised of 21 females and 7 males with a mean age of 61.1 years ±10.7 years; C-reactive protein was 7.0 mg/dl ± 8.59 mg/dl. In the RA group, 23 patients received nonsteroidal anti-inflammatory drugs, 22 received glucocorticoids, 11 received methotrexate, 3 received sulfasalazine and 2 received biologicals. The OA group comprised of 31 females and 25 males with a mean age of 68.5 years ±9.2 years; C-reactive protein was 4.7 mg/dl ± 10.4 mg/dl. In the OA group, 45 patients received nonsteroidal anti-inflammatory drugs. All patients underwent elective knee joint replacement surgery, and they were informed about the purpose of the study and gave written consent. The study was approved by the Ethics Committee of the University of Regensburg.

### Animals

Male DBA/1 mice, 6–8 weeks old, were purchased from Janvier (Heverlee, Belgium). The mice were housed 10 animals per cage, had free access to standard laboratory chow and water ad libitum, and were maintained under a 12-hour light/dark cycle. Experiments were conducted according to institutional and governmental regulations for animal use and were approved. (Government of the Oberpfalz AZ 54–2532.1-42/11).

### Synovial fibroblast and tissue preparation

Synovial tissue samples from OA and RA were obtained immediately after opening the knee joint capsule, the preparation of which was described [15]. Pieces of synovial tissue of up to 9 cm^2^ were excised. One part of the tissue was cut, placed in protective freezing medium and stored at −80 °C until further use (Tissue Tek, Sakura Finetek, Zoeterwoude, The Netherlands). Another part was minced and treated with dispase I (Roche Diagnostics, Mannheim, Germany). Digestion was carried out for 1 h at 37 °C on a shaking platform. The resulting suspension was filtered (70 μm) and centrifuged at 300 g for 10 min. The pellet was then treated with erythrocyte lysis buffer (20.7 g NH_4_Cl, 1.97 g NH_4_HCO_3_, 0.09 g EDTA ad 1 l H_2_O) for 5 min and again centrifuged for 10 min at 300 g. The pellet was resuspended in RPMI-1640 (Sigma-Aldrich, St. Louis, MO, USA) with 10 % fetal calf serum (FCS).

### Stimulation of RA synovial fibroblasts

To study cytokine and MMP-3 production, cells were stimulated in 2 % serum-containing RPMI-1640 medium with respective compounds 5 h prior to addition of TNF (10 ng/ml final concentration). Cell culture supernatants were used for enzyme-linked immunosorbent assays (ELISAs) 24 h (cytokines) or 48 h (MMP-3) after addition of compounds.

### TNF, IL-6 and IL-8 ELISA

Tests were conducted as described by the supplier (BD OptEIA, BD Biosciences, Heidelberg, Germany). Supernatants from SF were diluted 1:13 (SF), 1:400 (primary synoviocytes) (for IL-6 and IL-8) or used undiluted (TNF) before use. Inter- and intra-assay coefficient of variation was below 10 %.

### Matrix metalloproteinase-3 (MMP-3) ELISA

A total of 100 μl of tissue culture supernatants were transferred into 96-well plates (NUNC, Langenselbold, Germany) for 3 h at 37 degrees. Supernatants were removed and 1 % bovine serum albumin (BSA) in phosphate-buffered saline (PBS) was added for 1 h at room temperature to block unspecific binding. Then, anti-MMP3 (ab52915, Abcam, Cambridge, UK) antibody was added for 1 h at room temperature (1:1000, diluted in PBS with 1 % BSA). After washing, secondary antibody (1:2000, goat anti-rabbit poly-horseradish peroxidase (HRP), Fisher Scientific, Schwerte, Germany) was added for 1 h at room temperature. Standard MMP-3 protein was obtained from R&D Systems (Wiesbaden, Germany).

### Monitoring adhesion with the xCELLigence system

This system has been previously described [16]. For adhesion experiments, E-plates (Roche Diagnostics, Mannheim, Germany) were coated for 1 h at room temperature with 100 μl 10 μg/ml fibronectin (BD Biosciences, Heidelberg, Germany) in PBS. To block unspecific binding, 1 % BSA in PBS was added for 30 min. A total of 5000 pretreated cells (see above) were added to the E-plates and xCELLigence was programmed to monitor adhesion every minute for 240 min. After 60 min of incubation, adhesion remained linear so that this time point was chosen as the endpoint of adhesion measurement. Adhesion was quantified by averaging data from 1 min to 60 min of incubation. Untreated cells served as control and this value was set to 100 %.

### Immunocytochemistry

The following antibodies (all from Abcam, Cambridge, UK and Serotec (FAAH), Puchheim, Germany) were used: CB_1_ (ab23703, 30 μg/ml), CB_2_ (30 μg/ml, ab3561), TRPV1 (ab63083, 1:300), TRPA1 (ab62053, 30 μg/ml), COX-2 (ab62331, 30 μg/ml), and FAAH (MCA3101Z, 30 μg/ml). Cells were fixed with 3.7 % formaldehyde and permeabilized with 0.1 % Triton-X 100 in PBS. Slides were blocked with 1 % BSA in PBS/0.1 % Triton-X and incubated with primary antibody 3 h at 37 °C. Cells were washed and incubated with secondary cyanine-conjugated goat anti-rabbit antibody (1:500, Cy™3, Jackson Immunoresearch, West Grove, PA, USA) overnight at 4 °C. Isotype immunoglobulin G (IgG) or rabbit serum (TRPV1) served as negative control.

### Western blotting

The following antibodies (all from Abcam, Cambridge, UK and Serotec (FAAH), Puchheim, Germany) were used: CB_1_ (ab23703, 0.5 μg/ml), CB_2_ (ab3561, 1 μg/ml), TRPV1 (ab111973, 1 μg/ml), COX-2 (ab62331, 0.4 μg/ml), FAAH (serotec, MCA3101Z, 1 μg/ml), superoxide dismutase 2 (SOD-2) (Lab Frontier, Seoul, Korea, LF-PA0021, 1:2000), poly(ADP-ribose) polymerase (PARP) and glyceraldehyde 3-phosphate dehydrogenase (GAPDH) (Cell Signaling, Leiden, Netherlands, #9542 and #2118, 1:1000). A total of 1,000,000 **c**ells were lysed with RIPA buffer (10 mM Tris-Cl (pH 8.0), 1 mM EDTA, 0.5 mM EGTA, 1 % Triton X-100, 0.1 % sodium deoxycholate, 0.1 % SDS, 140 mM NaCl, complete protease inhibitor (Roche Diagnostics, Mannheim, Germany)) and protein content was determined. Gels (separation gel: 15 % acrylamide) were loaded with 20–50 μg protein and run for 60 min at 20 mA (Bio-Rad Laboratories, Puchheim, Germany). Gels were blotted at 80 V for 90 min on PVDF membranes (Bio-Rad Laboratories, Puchheim, Germany). Gels were blocked with 5 % nonfat dried milk in Tris-buffered saline (TBS) for 1 h. Primary antibodies were added overnight at 4 °C, whereas detection antibodies (donkey anti-rabbit IgG HRP (Dianova, Hamburg, Germany) 711-035-152, 1:5000)) were added afterwards for 1 h at room temperature. Signal was detected by ECL Prime (GE Healthcare, Freiburg, Germany) and analyzed in a V3 Western Workflow (Bio-Rad Laboratories, Puchheim, Germany).

### Proteom profiling (detection of phosphorylation status of selected MAP kinases)

For the assessment of MAP kinase activation we employed the proteome profiler human phospho-MAPK array kit (R&D Systems, Minneapolis, MN, USA, ARY001B). This assay is a membrane-based sandwich immunoassay and can detect 24 kinases simultaneously. The assay was conducted as described by the supplier.

### Cell-based ELISA

This test was used to quantify protein levels of CB_1_, CB_2_, TRPV1 and TRPA1. For antibodies used see the immunocytochemistry section. Final concentrations employed in the assay were 1.5 μg/ml for CB_1_, CB_2_ and TRPA1 (TRPV1, 1:1000). Cells were fixed with 3.7 % formaldehyde and permeabilized with 0.1 % Triton-X 100 in PBS. Microtiter plates were blocked with 1 % BSA in PBS/0.1 % Triton-X and incubated with primary antibody 3 h at 37 °C. Cells were washed and incubated with secondary poly HRP-conjugated goat anti-rabbit antibody (#32260, 1:5000, Thermo Fisher Scientific, Waltham, MA, USA) for 2 h at room temperature. Staining was visualized with 1-step Ultra TMB (#34029, Thermo Fisher Scientific).

### Induction of arthritis and scoring

Experiments were performed as previously described by our group [17]. Briefly, on day 0, mice were immunized intradermally at the base of the tail with 100 μg of bovine collagen type II (Chondrex, Redmond, WA, USA) emulsified in an equal volume of Freund’s complete adjuvant (Sigma-Aldrich, Deisenhofen, Germany). Each limb was graded separately, and points were given according to various inflamed regions: toes (0.5–2.5), midpaw (0.5–2.5), and wrist/ankle (0.5–2.5). Thus, a maximum total score of 15 points per extremity and 60 points per animal was given.

### Treatment of animals

Treatment was started when animals reached a score of four points. Daily injections of either FAAH inhibitor JNJ1661010 (R&D Systems, Wiesbaden, Germany) or vehicle were given intraperitoneally (i.p.) JNJ1661010 was dissolved in 20 % Pharmasolve (N-methyl-2-pyrrolidone; Sigma-Aldrich, Taufkirchen, Germany), 5 % cremophor (Sigma-Aldrich, Taufkirchen, Germany) and 75 % PBS.

### Reagents

Anandamide, oleoylethanolamide, palmitoylethanolamide, N-phenyl-4-(3-phenyl-1,2,4-thiadiazol-5-yl)-1-piperazinecarboxamide (JNJ1661010), cyclohexylcarbamic acid 3'-(Aminocarbonyl)-[1,1'-biphenyl]-3-yl ester (URB597), 5-[(1,1'-Biphenyl]-4-yl)methyl]-N*,*N-dimethyl-1H-tetrazole-1-carboxamide (Ly2183240), N-(4-nitro-2-phenoxyphenyl)methanesulfonamide (nimesulide), 1-[8-(2-chlorophenyl)-9-(4-chlorophenyl)-9H-purin-6-yl]-4-(ethylamino)piperidine-4-carboxamide hydrochloride (CP945598), *N*-(1,3-benzodioxol-5-ylmethyl)-1,2-dihydro-7-methoxy-2-oxo-8-(pentyloxy)-3-quinolinecarboxamide (JTE-907), *N*-[2-(4-chlorophenyl)ethyl]-1,3,4,5-tetrahydro-7,8-dihydroxy-2*H*-2-benzazepine-2-carbothioamide (capsazepine), 6,7-deepoxy-6,7-didehydro-5-deoxy-21-dephenyl-21-(phenylmethyl)-daphnetoxin,20-(4-hydroxy-5-iodo-3-methoxybenzeneacetate) (5’-iodoresiniferatoxin), (1*E*,3*E*)-1-(4-fluorophenyl)-2-methyl-1-pentene-3-one oxime (A967079) were obtained from R&D Systems/Tocris (Wiesbaden, Germany). Concentrations of respective drugs are given in each figure. A967079, capsazepine, nimesulide, JNJ1661010, CP945598, JTE-907 and 5’-iodoresiniferatoxin were used in concentrations that guaranteed maximal inhibition of the specified target protein. These data were either gathered from experiments described in the literature, already published EC_50_ data or our own previous experiments. AEA, OEA and PEA were used from 10^−12^/10^−11^ M to 10^−6^ M since these compounds bind a wide variety of target receptors with different affinities and a bell-shaped curve was therefore expected.

### Statistical analysis

Statistical analysis was performed with SigmaPlot 12 (Systat Software Inc., San Jose, CA, USA) and SPSS 20 (IBM, Armonk, NY, USA). The statistical tests used are given in the figure legends. Statistical significance was obtained with *p* values <0.05.

## Results

### Anandamide reduces IL-6, IL-8 and TNF production by primary mixed synoviocytes

In the first experiments, the effects of AEA with or without FAAH inhibition on cytokine production by primary mixed synovial cells containing fibroblasts, macrophages, T and B lymphocytes and dendritic cells under normoxic (20 % O_2_) serum-free conditions were determined. Synoviocytes from donors with osteoarthritis (OA) served as a nonchronic inflammatory control.

**Fig. 1:**
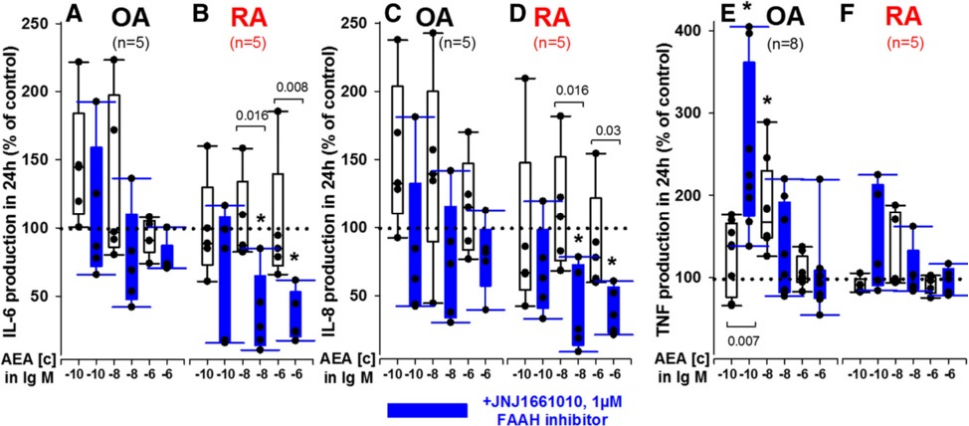
Influence of anandamide (AEA) with or without fatty acid amide hydrolase (FAAH) inhibition. Effects are described on IL-6 (**a**, **b**), IL-8 (**c**, **d**) and TNF (**e**, **f**) production in RA mixed synovial cell cultures. Cytokine production was determined under normoxic (20 % O_2_) conditions. *p <0.01 vs. control (100 %). Mann-Whitney *U* test was used for comparisons between groups; paired *t* test was used for comparisons vs. control. All data are given as box and vertical dot blots. The boundary of the box closest to zero indicates the 25^th^ percentile, the line within each box indicates the median and the upper boundary of the box the 75^th^ percentile. Error bars below and above the box indicate the 10th and 90th percentiles. *Blue color* represents treatment with the FAAH inhibitor JNJ1661010 (1 μM). *IL-6* interleukin-6, *IL-8* interleukin-8, *RA* rheumatoid arthritis, *TNF* tumor necrosis factor

Under these conditions, AEA (at 10^−6^ M and 10^−8^ M) and concomitant FAAH inhibition with JNJ1661010 (1 μM) reduced the production of IL-6 and IL-8 by RA but not OA mixed synoviocytes (Fig. 1b, d). TNF production was augmented only by OA synoviocytes (at 10^−8^ M without FAAH inhibition and 10^−10^ M with FAAH inhibition) (Fig. 1e). Average control values for cytokines (dotted line at 100 % in Fig. 1) were 110 ± 44 ng/ml (OA) and 148 ± 49 ng/ml (RA) for IL-6, 103 ± 49 ng/ml (OA) and 171 ± 56 ng/ml (RA) for IL-8, and 66 ± 68 pg/ml (OA) and 163 ± 166 pg/ml (RA) for TNF.

### Synovial fibroblasts express several receptors and enzymes involved in endocannabinoid action

**Fig. 2:**
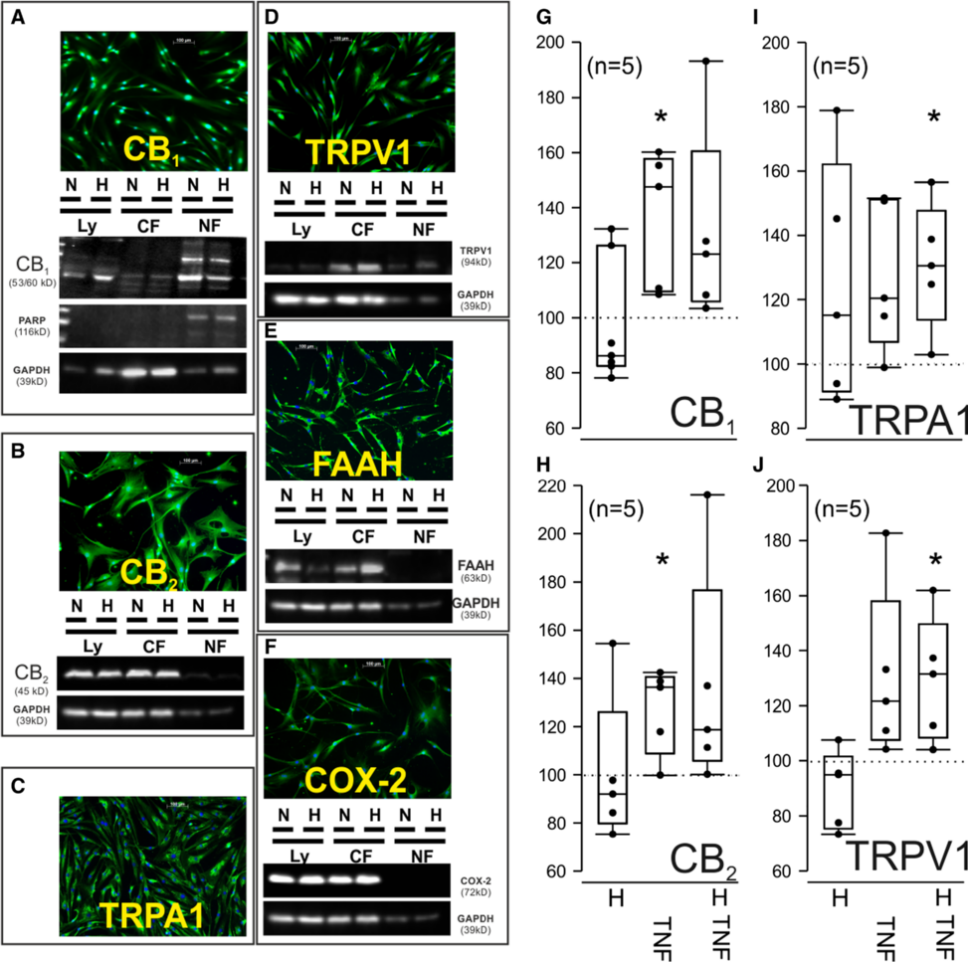
N-acylethanolamine binding receptors visualized by western blotting and immunofluorescence and quantification of CB_1_, CB_2_, TRPV1 and TRPA1 in response to hypoxia and TNF in RASF by cell-based ELISA. Immunofluorescent staining and western blotting revealed the cellular localization of CB_1_ (**a**), CB_2_ (**b**), TRPA1 (**c**), TRPV1 (**d**), FAAH (**e**) and COX-2 (**f**) protein. Original magnification is 400×. Quantification of CB_1_ (**g**), CB_2_ (**h**), TRPA1 (**i**) and TRPV1 (**j**) protein by cell-based ELISA after culture of RASF for 48 h under either hypoxia, TNF stimulation (10 ng/ml) or hypoxia combined with TNF (10 ng/ml) in RASF. g-j: Data are given as % of unstimulated control RASF. *p <0.05 vs. normoxic control (*dotted line*). Paired Wilcoxon test was used for comparisons. *LY* = complete cell lysate, *CF* = cytosolic fraction, *NF* = nuclear fraction, *N* = normoxia, *H* = hypoxia, *PARP* = poly(ADP-ribose)-polymerase 1 (control for nuclear localization), *GAPDH* = glycerinaldehyd-3-phosphat-dehydrogenase (control for cytosolic localization). *CB*_*1*_ cannabinoid receptor type 1, *CB*_*2*_ cannabinoid receptor type 2, *COX-2* cyclooxygenase-2, *ELISA* enzyme-linked immunosorbent assay, *FAAH* fatty acid amide hydrolase*, IL-6* interleukin 6, *IL-8* interleukin 8, *MMP-3* matrix metalloproteinase 3 (stromelysin), *RA* rheumatoid arthritis, *SF* synovial fibroblast(s), *TNF* tumor necrosis factor, *TRPA1* transient receptor potential ankyrin 1, *TRPV1* Transient receptor potential vanilloid channel

In subsequent experiments, RASF generated from primary synovial cells were investigated, since effects of AEA were only augmented in mixed RA synoviocytes (see above). AEA, PEA and OEA bind a wide array of receptors and enzymes and, therefore, expression of possible target proteins was assessed in RASF (Fig. 2). Immunocytochemistry and western blotting (not for TRPA1, since antibodies were not suitable for western blotting) revealed that RASF not only express CB_1_, CB_2,_ FAAH and COX-2 but also TRPV1 and TRPA1 (Fig. 2a-f) (see also Figure S1 in Additional file 1 for entire blots and blocking peptide control). Surprisingly, CB_1_ was found located exclusively in the nuclear membrane (Fig. 2a). Addition of TNF (10 ng/ml, 48 h) and hypoxia influenced protein levels of CB_1_, CB_2_, TRPV1 and TRPA1 (Fig. 2g-j). CB_1_ and CB_2_ protein were increased by TNF treatment (Fig. 2g, h). TRPV1 and TRPA1 were significantly increased in RASF when hypoxia was combined with TNF treatment (Fig. 2i, j).

### Anandamide reduces TNF-induced IL-6, IL-8 and MMP-3 production by RASF in a TRPA1-dependent manner

SF contribute not only to erosions in RA but also fuel inflammation by producing large amounts of cytokines and chemokines [18]. MMP-3 production served as an additional read-out because this MMP is abundantly produced by SF upon cytokine challenge and is a major contributor to erosions in RA [19]. All experiments with isolated SF were conducted under hypoxic conditions (1 % O_2_), since this represents the oxygen partial pressure in synovial tissue [4].

**Fig. 3:**
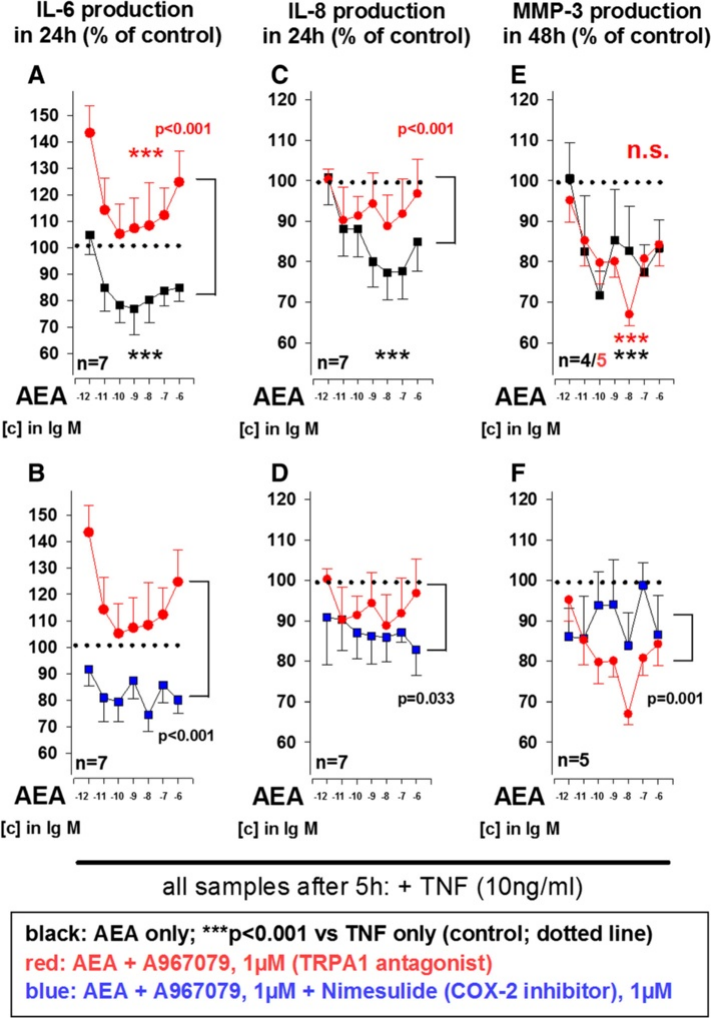
Influence of anandamide (AEA) on TNF-induced (10 ng/ml) IL-6 (**a**, **b**), IL-8 (**c**, **d**) and MMP-3 (**e**, **f**) production by RASF under hypoxic conditions. The *dotted line* indicates the control level of 100 % (TNF without AEA). ***p <0.001, for comparisons vs. control; *p* values for comparisons between AEA/A967079 and AEA/A967079/nimesulide are given in the graph. n.s., not significant. The general linear model with Dunnett’s post hoc test was used for all comparisons. All data are given as mean ± SEM. Cyclooxygenase-2 inhibitor = nimesulide, TRPA1 antagonist = A967079. *IL-6* interleukin 6, *IL-8* interleukin 8, *MMP-3* matrix metalloproteinase 3 (stromelysin), *RA* rheumatoid arthritis, *SF* synovial fibroblast(s), *TNF* tumor necrosis factor, *TRPA1* transient receptor potential ankyrin 1, *TRPV1* transient receptor potential vanilloid channel

In the presence of TNF (10 ng/ml), AEA preincubation (5 h, 10^−6^ M to 10^−12^ M) significantly reduced IL-6, IL-8 and MMP-3 production by RASF (Fig. 3a, c, e). The effects of AEA on TNF-induced IL-6, IL-8 and MMP-3 production were not abrogated by CB_1_, CB_2_ or TRPV1 antagonism (Figure S2 in Additional file 2). However, the TRPA1 antagonist A967079 (10^−6^ M) blocked the inhibitory effects of AEA on TNF-induced IL-6 (Fig. 3a) and IL-8 (Fig. 3c) but not on MMP-3 production (Fig. 3e) in RASF. The effect of AEA together with A967079 on IL-6 and IL-8 production were inhibited when combined with the COX-2 inhibitor nimesulide (10^−6^ M) (Fig. 3b, d). MMP-3 production by RASFs was significantly reduced when A967079 was combined with AEA (Fig. 3e). This effect was attenuated by the addition of nimesulide (Fig. 3f). Since inhibition of FAAH and the putative AEA transporter enhance the effects of AEA [20, 21], we also combined AEA with the dual FAAH inhibitor/AEA transport inhibitor Ly2183240 (10^−7^ M). However, in contrast to mixed synovial cell cultures, the effects of AEA on TNF-induced cytokine production were not enhanced by FAAH inhibition in RASF (data not shown). Average control values (*dotted line* at 100 % in Fig. 3) were 3.82 ± 2.68 ng/ml for IL-6, 3.60 ± 1.58 ng/ml for IL-8 and 42 ± 17 ng/ml for MMP-3.

### Anti-inflammatory activity of N-acylethanolamines PEA and OEA are also mediated by TRP channels

**Fig. 4:**
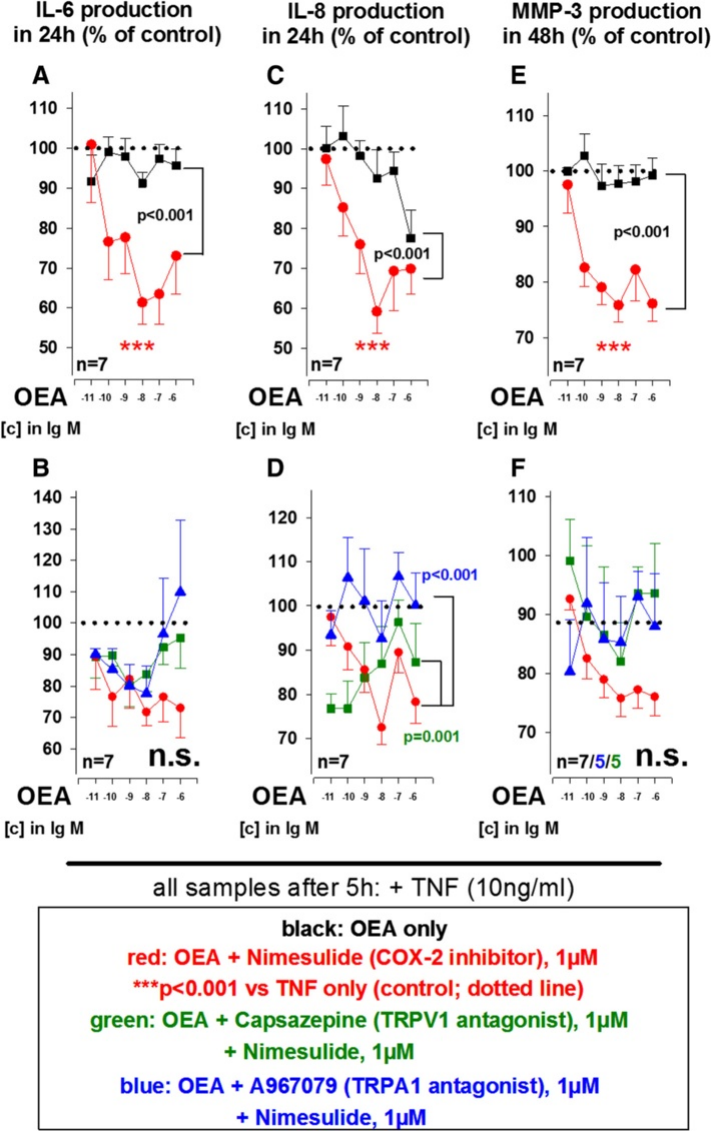
Influence of oleoylethanolamine (OEA) on TNF-induced IL-6 (**a**, **b**), IL-8 (**c**, **d**) and MMP-3 (**e**, **f**) production by RASF under hypoxic conditions. The *dotted line* indicates the control level of 100 % (TNF alone). ***p <0.001 for comparisons vs. control (TNF alone); *p* values for comparisons between OEA/A967079 and OEA/A967079/nimesulide or OEA/capsazepine/nimesulide are given in the graph. n.s., not significant. The general linear model with Dunnett’s post hoc test was used for all comparisons. All data are given as mean ± SEM. TRPV1 antagonist = capsazepine, TRPA1 antagonist = A967079, cyclooxygenase-2 inhibitor = nimesulide. *IL-6* interleukin 6, *IL-8* interleukin 8, *MMP-3* matrix metalloproteinase 3 (stromelysin), *RA* rheumatoid arthritis, *SF* synovial fibroblast(s), *TNF* tumor necrosis factor, *TRPA1* transient receptor potential ankyrin 1, *TRPV1* transient receptor potential vanilloid channel

The AEA congeners OEA and PEA are also activators/desensitizers at TRPV1 channels and we therefore tested whether cation channels might be general targets of N-acylethanolamines in SF. While OEA (10^−11^ M to 10^−6^ M) by itself did not modulate IL-6, IL-8 and MMP-3 production (Fig. 4a, c, e, *black curves*), combination with the COX-2 inhibitor nimesulide significantly reduced cytokine and MMP-3 production (Fig. 4a, c, e, *red curves*). A combination of OEA and the FAAH inhibitor JNJ1661010 (100nM) had no effect on IL-6, IL-8 and MMP-3 secretion by RASF (data not shown). Similar to its effects in combination with AEA, the TRPA1 antagonist A967079 (1 μM) blocked the effects of nimesulide/OEA on TNF-induced IL-8 production (Fig. 4e, blue curve). In addition, the TRPV1 anatgonist capsazepine (1 μM) together with nimesulide/OEA also modulated IL-8 production by RASF (Fig. 4d).

**Fig. 5:**
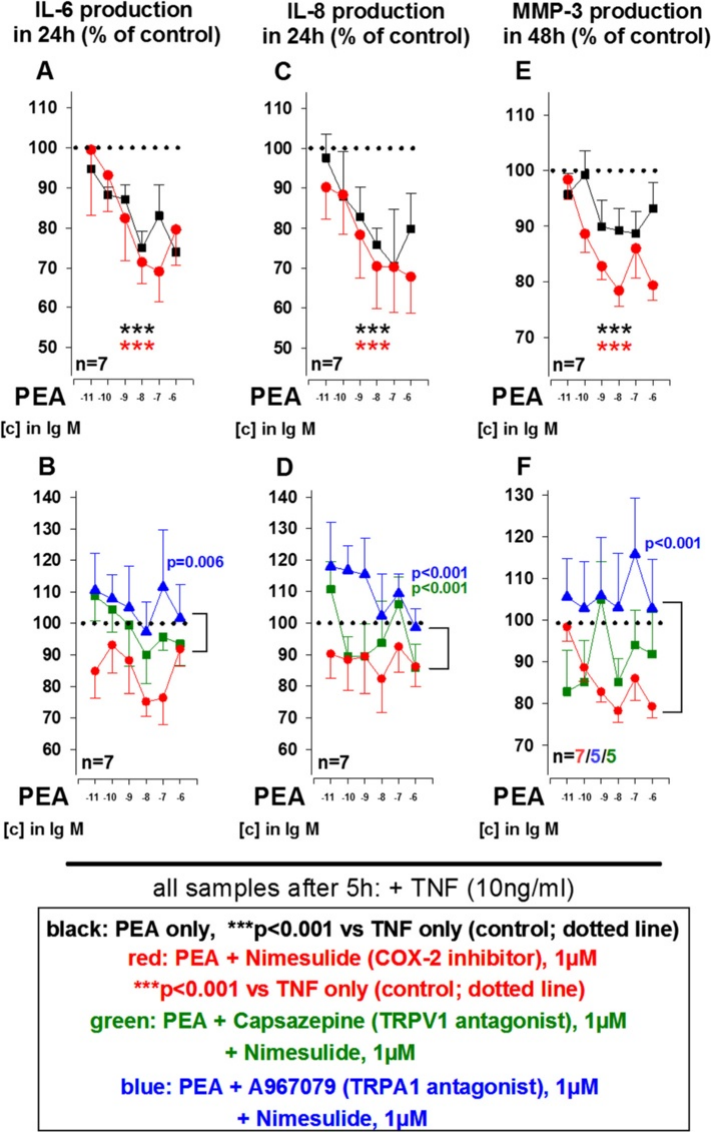
Influence of palmitoylethanolamine (PEA) on TNF-induced IL-6 (**a**, **b**), IL-8 (**c**, **d**) and MMP-3 (**e**, **f**) production by RASF under hypoxic conditions. The *dotted line* indicates the control level of 100 % (TNF alone). ***p <0.001 for comparisons vs. control (TNF alone); *p* values for comparisons between PEA/A967079 and PEA/A967079/nimesulide or PEA/capsazepine/nimesulide are given in the graph. n.s., not significant. The general linear model with Dunnett’s post hoc test was used for all comparisons. All data are given as mean ± SEM. TRPV1 antagonist = capsazepine, TRPA1 antagonist = A967079, cyclooxygenase-2 inhibitor = nimesulide. *IL-6* interleukin 6, *IL-8* interleukin 8, *MMP-3* matrix metalloproteinase 3 (stromelysin), *RA* rheumatoid arthritis, *SF* synovial fibroblast(s), *TNF* tumor necrosis factor, *TRPA1* transient receptor potential ankyrin 1, *TRPV1* transient receptor potential vanilloid channel

PEA (10^−11^ M to 10^−6^ M) alone significantly reduced IL-6 and IL-8 secretion by RASF (Fig. 5a, c, e). FAAH inhibition did not increase but slightly attenuated effects of PEA on IL-8 production by RASF (data not shown). In contrast to OEA, COX-2 inhibition by nimesulide did not enhance the effects of PEA on IL-6, IL-8 and MMP-3 production (Fig. 5a, c, e, *red curves*). However, when nimesulide and PEA were combined with the TRPA1 inhibitor A967079 (1 μM), anti-inflammatory effects of PEA/nimesulide on IL-6, IL-8 and MMP-3 were inhibited (Fig. 5b, d, f, *blue curves*). Furthermore, TRPV1 antagonism by capsazepine (1 μM) attenuated effects of PEA/nimesulide on IL-8 production (Fig. 5d, *green curve*). Nimesulide, capsazepine and A967079 alone had no effect on RASF cytokine and MMP-3 production.

### Endocannabinoids regulate intracellular signaling in SF

**Fig. 6:**
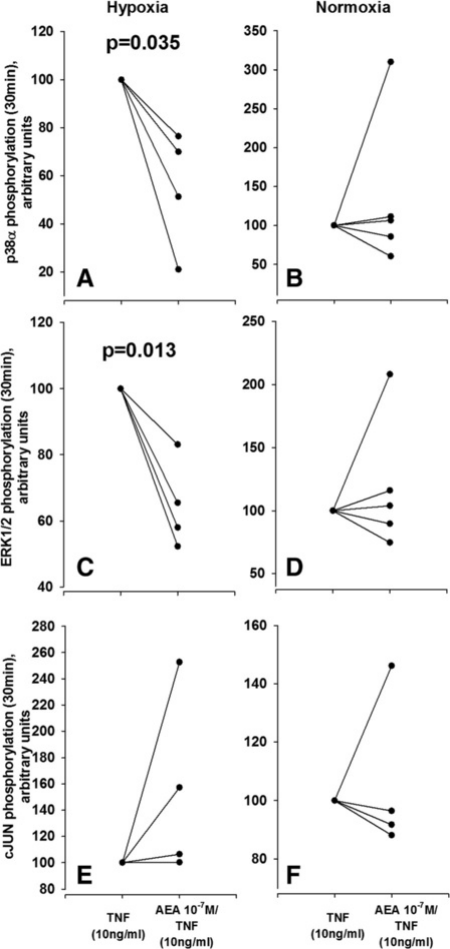
Impact of anandamide on mitogen-activated protein kinase pathways in RASF. **a-f** Analysis of p38α phosphorylation (**a**, **b**), ERK1/2 phosphorylation (**c**, **d**) and cJun phosphorylation (**e**, **f**) under TNF treatment with or without AEA pretreatment under normoxic (**a**, **c**, **e**) and hypoxic (**b**, **d**, **f**) conditions. Paired *t* test was used for comparisons. Number of patients included was n = 4 (**a**, **c**, **e**) and n = 5 (**b**, **d**, **f**). *AEA* anandamide, *RA* rheumatoid arthritis, *SF* synovial fibroblast(s), *TNF* tumor necrosis factor

To address the question how EC modulate proinflammatory cytokine signaling, proteome profiling was employed to detect changes in phosphorylation of proinflammatory intracellular signaling proteins. Preincubation of SF with AEA 5 h prior to TNF (10 ng/ml) treatment significantly reduced phosphorylation of p38α (Fig. 6a) and ERK1/2 (Fig. 6c) but not cJUN (Fig. 6e) in RASF. This only occurred under hypoxic but not normoxic conditions (Fig. 6b, d).

### Influence of anandamide on proliferation of OASF and RASF

**Fig. 7:**
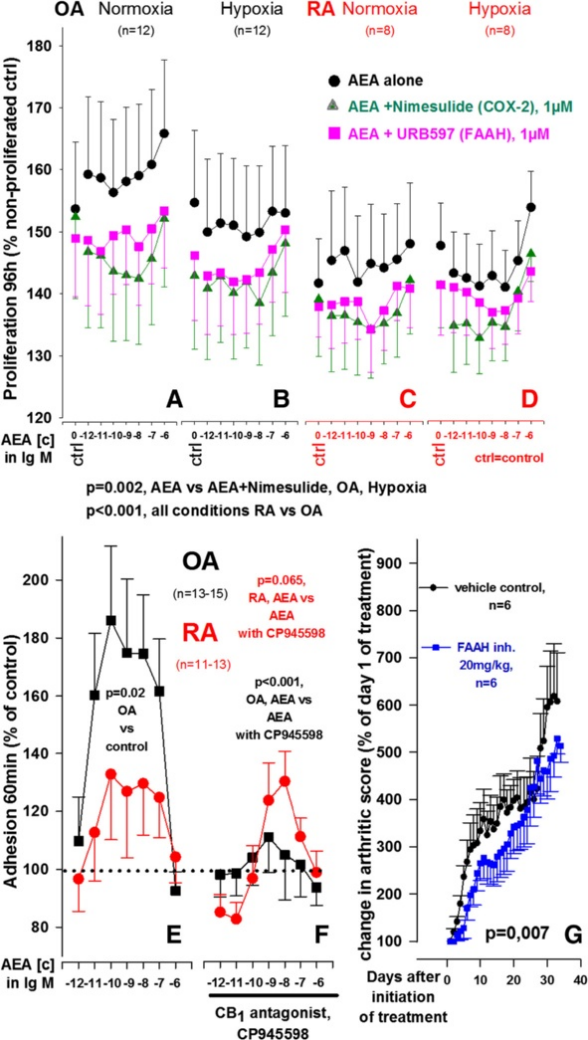
Influence of anandamide (AEA) on proliferation and adhesion of OASF and RASF and collagen-induced arthritis in mice. (**a**-**d**) Impact of AEA on OASF (**a**, **b**) and RASF (**c**, **d**) proliferation under normoxic (**a**, **c**) and hypoxic (**b**, **d**) conditions. **e**, **f** Adhesion of RASF and OASF to fibronectin after 24 h treatment with AEA (**e**) or with AEA + CP945598 (**f**) under normoxic conditions. **g** Impact of FAAH inhibition on collagen-induced arthritis in mice. The general linear model with Dunnett’s post hoc test was used for all comparisons. All data are given as mean ± SEM. Nimesulide = cyclooxygenase-2 inhibitor, URB597 = covalent, irreversible FAAH inhibitor, CB_1_ antagonist = CP945598 =, covalent, reversible FAAH inhibitor = JNJ1661010. All antagonists/inhibitors (**a**-**f**) were used at [c] = 1 μM. *FAAH* fatty acid amide hydrolase, *OA* osteoarthritis, *RA* rheumatoid arthritis, *SF* synovial fibroblast(s)

Hyperproliferation and resistance to apoptosis are two characteristics of RASF that enhance cartilage destruction [2]. Therefore, the ability of AEA to reduce SF proliferation over 96 h was assessed. Since OASF and RASF proliferate differently (own observations), we included OASF as control group in proliferation studies. Proliferation was conducted for 4 days under 10 % serum conditions, and therefore URB597 (1 μM) was used, since this compound guarantees prolonged FAAH inhibition. AEA alone or in combination with the FAAH inhibitor URB597 or the COX-2 inhibitor nimesulide did not significantly modulate proliferation of RASF and OASF (Fig. 7a-d). However, under hypoxic conditions, OASF proliferation was significantly decreased when AEA was combined with nimesulide when compared with AEA alone (Fig. 7b). Furthermore RASF proliferated significantly slower than OASF.

### Anandamide supports adhesion of OASF and RASF to fibronectin in a CB_1_-dependent manner

Our group already demonstrated the involvement of CB_1_ and CB_2_ in the adhesion of OASF and RASF to fibronectin [16] and therefore the influence of AEA on this functional parameter was determined. AEA significantly increased adhesion to fibronectin over a large dose range (10^−12^ M to 10^−6^ M) in OASF but did not reach significance in RASF (Fig. 7e). In OASF with a trend in RASF, this effect was significantly reduced by the CB_1_ antagonist CP945598 (Fig. 7f) but not by the CB_2_ antagonist JTE907 (1 μM) or the TRPV1 antagonist capsazepine (1 μM) (not shown).

### Inhibition of anandamide degradation ameliorates collagen-induced arthritis in mice

AEA reduced several inflammatory mediators and therefore the influence of FAAH inhibition on experimental arthritis was investigated. Mice were injected daily with JNJ1661010, a potent and selective inhibitor of FAAH and treatment reduced arthritis score in comparison to control animals (Fig. 7g).

## Discussion

For the first time, it is demonstrated that N-acylethanolamines reduce the production of inflammatory mediators by OA and RA mixed synovial cells and RASF and that AEA alters SF adhesion. Proliferation was not altered by AEA itself but was influenced when AEA was combined with nimesulide. These effects are dependent on activation of the TRPA1/TRPV1 channels and COX-2. A possible anti-inflammatory mechanism involves downregulation of MAP kinase signaling (p38α, ERK1/2) by AEA. Furthermore it is shown that elevation of EC levels by inhibiting their degradation is beneficial in collagen-induced arthritis of the mouse.

This study demonstrated mixed synovial cell cytokine production was altered by AEA only when FAAH was inhibited suggesting high AEA turnover by synoviocytes. Reduction of lymphocyte and macrophage FAAH expression strongly increases AEA concentrations and certain threshold AEA concentrations might be necessary to elicit anti-inflammatory effects [22].

Before evaluating the effects of AEA on RASF, screening for possible target receptors was conducted. TRPV1 protein was detected in RASF and stimulation increased the production of the proinflammatory cytokine IL-6 [23]. Furthermore, expression and function of several other TRP channels have been demonstrated in RASF [24].

In addition, expression of CB_1_ and CB_2_ was demonstrated in SF but the cellular localization was not investigated [6]. This study revealed that CB_2_ is abundantly expressed and embedded in the cell membrane whereas CB_1_ is located almost exclusively at the nuclear membrane suggesting a specialized role of CB_1_ in SF. Nuclear CB_1_ has a unique role in mobilizing calcium from internal stores, which was revealed by intracellular administration of AEA [25].

All our studies with isolated SF regarding cytokine and MMP-3 production have been conducted under hypoxia (1 % O_2_), which reflects changes in O_2_ partial pressure during chronic synovial inflammation [4].

The inhibitory effects of AEA and PEA on IL-8 production in RASF were partially reversed by FAAH inhibition. In addition, COX-2 inhibition enhanced the effects of the otherwise inactive compound OEA. This suggests an intracellular mechanism of action and demonstrates that prolonged activity of parent compounds might have detrimental effects. While AEA, PEA and OEA are all substrates for FAAH, only AEA is hydrolyzed by COX-2 yielding possible proinflammatory metabolites [26]. This feature is important, since we demonstrated constitutively high protein levels of COX-2 in SF. Therefore, AEA but not PEA or OEA metabolism might be redirected to COX-2 and degradation products from this reaction might reduce the anti-inflammatory effects of AEA.

We found the effects of AEA, PEA and OEA to be dependent on TRPA1 and TRPV1 modulation and activation of these channels have been connected to pro- and anti-inflammatory effects depending on cell type and setting [27]. The N-acylethanolamines OEA, PEA and AEA have been described to bind and activate TRPV1, but only AEA weakly activates TRPA1 in high concentrations [28].

Importantly, our study revealed that the effects of all three N-acylethanolamines were blocked by TRPA1 antagonism and partly by TRPV1 antagonism. TRPA1 is upregulated by hypoxia and its calcium-gating capabilities are enhanced under these conditions [5, 29]. Furthermore, the activity of TRPA1 is regulated by TRPV1 and possibly vice versa since there is extensive crosstalk between these two channels via calcium [30]. In addition, presence of both channels in one cell limits constitutive activity and calcium leakage, while presence of only TRPA1 increases intracellular calcium with detrimental effects on cellular health [30].

In our own experiments TNF was used as proinflammatory stimulus to initiate cytokine production. This cytokine not only enhances TRPA1 expression but also increases COX-2 expression and initiates prostaglandin synthesis in SF [31]. This leads to a protein kinase C-dependent phosphorylation and sensitization of TRPV1 [32]. Given the strong interaction between TRPV1 and TRPA1, it is likely that TNF also sensitizes TRPA1 in SF via COX-2. Inhibition of TRPA1 with concomitant AEA, PEA or OEA stimulation might block the desensitizing capacity of N-acylethanolamines on TRPV1 and activation of TRPV1 by capsaicin increases production of IL-6 [23].

Interestingly, COX-2 inhibition prevented the proinflammatory effects of TRPA1 antagonism with concomitant AEA stimulation. In addition, the anti-inflammatory effects of OEA and PEA combined with COX-2 inhibition were blocked by TRPA1 antagonism. This suggests that COX-2-derived prostaglandins might counteract the desensitizing effects of AEA, OEA and PEA on TRPV1/TRPA1. In the case of AEA, COX-2 degradation products (prostamides) might also influence TRPV1 or TRPA1 activity.

A possible mechanism for the anti-inflammatory effects of AEA is modulation of MAP kinase signaling. In this study, it was demonstrated that AEA reduces TNF-induced p38α and ERK1/2 phosphorylation. This phenomenon has already been described in microglial cells, where AEA increases the expression of MKP-1, a phosphatase and negative regulator of MAP kinase signaling [33].

AEA has antiproliferative effects and this propensity of cannabinoids has been investigated in several cell types and diseases [34]. In this study AEA did not significantly modulate proliferation of SF. It might be that AEA only elicits antiproliferative effects during an inflammatory challenge and since no cytokines were added in our own experiments no effects were observed.

In previous studies, CB_1_/CB_2_ activation increased integrin-driven adhesion [16]. AEA treatment of SF resulted in a bell-shaped dose–response curve, which might be due to increased activation of TRPV1/TRPA1 at high AEA levels leading to increased Ca^2+^ mobilization and decreased integrin affinity. At low levels, AEA only activates CB_1_ and, thus, Ca^2+^ mobilization would be optimal to activate integrins. The observed differences in adhesion between OASF and RASF in response to AEA may have several reasons. Although we did not detect differences in CB_1_, CB_2_ or TRPV1 protein levels, intracellular signaling might be altered in RASF compared to OASF or healthy SF. Epigenetic studies already revealed several differences between “transformed” RASF and healthy SF [35]. Consequently, RASF retain their invasive and migrative phenotype when implanted in SCID mice, whereas OASF do not demonstrate this feature [3]. As adhesion precedes invasion and migration, the observed differences between OASF and RASF might therefore also be due to epigenetic alterations. These changes might manifest in differential endocannabinoid signaling in OASF and RASF. This is further emphasized by a different composition of endocannabinoids in healthy synovial fluid and synovial fluid from RA patients [6].

Our in vitro findings regarding anti-inflammatory effects of AEA were also studied in the CIA model. Arthritic mice benefited when treated with the FAAH inhibitor JNJ1661010. FAAH inhibition raises EC levels in most organs but the magnitude and composition of EC is dependent on the FAAH inhibitor used [36]. Kinsey et al. used the FAAH inhibitor URB597 for the treatment of CIA and found beneficial effects, which were dependent on activation of CB_2_ [37]. The effects in the present study were subtle and this likely depends on the FAAH inhibitor used. In addition, we used a dose (20 mg/kg) which was effective in decreasing pain in rats but higher doses might be needed in mice due to faster turnover or under systemic proinflammatory conditions like in collagen-induced arthritis. In contrast to JNJ1661010, URB597 also inhibits several other esterases, some of which might also contribute to N-acylethanolamine degradation [36].

## Conclusions

This study demonstrates that cytokine production of synoviocytes and RASF is modulated by the N-acylethanolamines AEA, PEA and OEA in a TRPV1/TRPA1-dependent manner. The effects of these compounds were enhanced by COX-2 inhibition suggesting a sensitizing role for COX-2-derived metabolites at TRPs. Furthermore, AEA reduced MAP kinase signaling and increased adhesion of SF to fibronectin. The beneficial effects of elevated EC levels were demonstrated in the CIA model in mice. These findings show that activation of the EC system can be beneficial in arthritis and manipulation of this system (especially by combination of a COX-2 and a FAAH inhibitor) might be a promising strategy to reduce erosions and inflammation in arthritis.

## Additional files

Additional file 1:
**Visualization of CB**_**1**_ **(A), CB**_**2**_ **(B) and TRPV1 (C) protein in RASF by western blot (whole membranes) and immunofluorescent staining of CB**_**1**_ **(D) with and without blocking peptide.** A, B and C are corresponding whole membranes from blots depicted in Fig. 2d CB_1_-specific antibody ab23703 was used with (*right panel*) and without blocking peptide (*left panel*). (TIFF 13914 kb) Additional file 2:
**Influence of CB**_**1**_ **(A, D, G), CB**_**2**_ **(B, E, H) or TRPV1 (C, F, I) antagonism together with anandamide (AEA) on TNF-induced (10 ng/ml) interleukin- 6 (IL-6) (A, B, C), IL-8 (D, E, F) and MMP-3 (G, H, I) production by RASF under hypoxic conditions.** The *dotted line* indicates the control level of 100 % (TNF without AEA). The general linear model with Dunnett’s post hoc test was used for all comparisons. All data are given as mean ± SEM. CB_1_ antagonist = CP945598, CB_2_ antagonist = JTE-907, TRPV1 antagonist = 5’iodoresiniferatoxin. (TIFF 681 kb)

## Abbreviations

2-AG: 2-Arachidonylglycerol
AEA: Arachidonylethanolamide, anandamide
BSA: Bovine serum albumin
CB_1_: Cannabinoid receptor type 1
CB_2_: Cannabinoid receptor type 2
CIA: Collagen type II-induced arthritis
COX-2: Cyclooxygenase-2
EC: Endocannabinoid
ELISA: Enzyme-linked immunosorbent assay
FAAH: Fatty acid amide hydrolase
FCS: Fetal calf serum
GAPDH: Glyceraldehyde 3-phosphate dehydrogenase
GPR18: G-protein coupled receptor 18
GPR55: G-protein coupled receptor 55
HRP: Horseradish peroxidase
IgG: Immunoglobulin G
IL-6: Interleukin 6
IL-8: Interleukin 8
MAGL: monoacylglycerol lipase
MAP: Mitogen-activated protein
MMP-3: Matrix metalloproteinase 3 (stromelysin)
OA: Osteoarthritis
OEA: Oleoylethanolamine
PARP: Poly(ADP-ribose) polymerase
PBS: Phosphate-buffered saline
PEA: Palmitoylethanolamine
RA: Rheumatoid arthritis
SF: Synovial fibroblast(s)
SOD-2: Superoxide dismutase 2
TBS: Tris-buffered saline
TNF: Tumor necrosis factor
TRPA1: Transient receptor potential ankyrin 1
TRPV1: Transient receptor potential vanilloid channel
