# Supplementary figures and images for: Anti-inflammatory effects of N-acylethanolamines in rheumatoid arthritis synovial cells are mediated by TRPV1 and TRPA1 in a COX-2 dependent manner
Source: Arthritis Res Ther. 2015 Nov 14;17:321. doi: 10.1186/s13075-015-0845-5 (PMC4644337; doi:10.1186/s13075-015-0845-5)

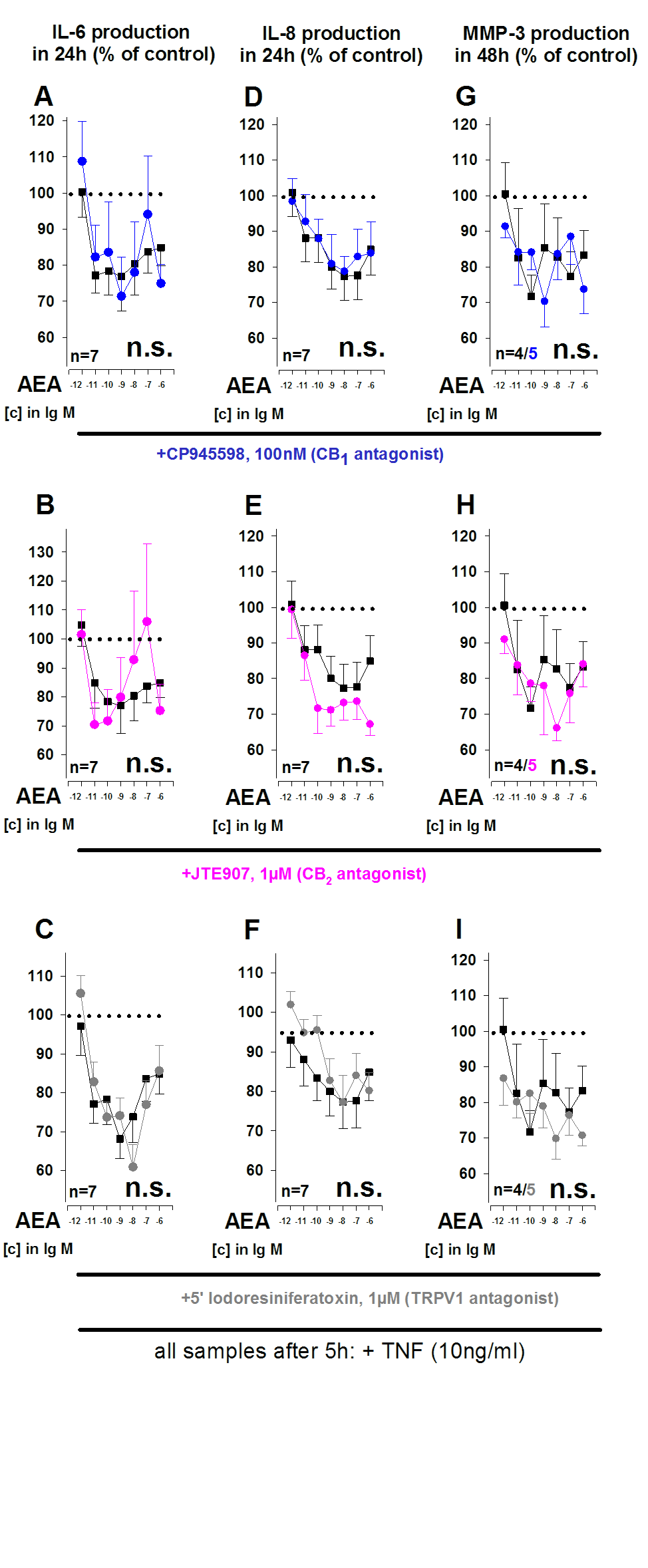

Supplement: Additional file 2: — Influence of CB 1 (A, D, G), CB 2 (B, E, H) or TRPV1 (C, F, I) antagonism together with anandamide (AEA) on TNF-induced (10 ng/ml) interleukin- 6 (IL-6) (A, B, C), IL-8 (D, E, F) and MMP-3 (G, H, I) production by RASF under hypoxic conditions. The dotted line indicates the control level of 100 % (TNF without AEA). The general linear model with Dunnett’s post hoc test was used for all comparisons. All data are given as mean ± SEM. CB1 antagonist = CP945598, CB2 antagonist = JTE-907, TRPV1 antagonist = 5’iodoresiniferatoxin. (TIFF 681 kb) [file 13075_2015_845_MOESM2_ESM.tiff]
